# Advanced Gastric Adenocarcinoma Presenting With Shoulder Pain and Diplopia

**DOI:** 10.7759/cureus.98409

**Published:** 2025-12-03

**Authors:** Omar Gandarilla, Aashi Saraf, Uqba Khan, Brandon Swed

**Affiliations:** 1 Medicine, NewYork-Presbyterian Brooklyn Methodist Hospital, New York, USA; 2 Medical Oncology, New York Presbyterian Hospital/Weill Cornell Medicine, New York, USA; 3 Medicine, New York Presbyterian Hospital/Weill Cornell Medicine, New York, USA

**Keywords:** advanced gastric cancer, diplopia, muscle metastasis, orbital metastasis, soft tissue metastasis

## Abstract

Gastric adenocarcinoma is a leading cause of cancer-related mortality worldwide, with many cases diagnosed at an advanced stage. While liver, peritoneal, and lymphatic involvement are common, metastases to soft tissue and orbital structures are exceedingly rare and may present with non-specific symptoms that mimic benign conditions. We report the case of a 48-year-old woman who presented with persistent musculoskeletal pain and diplopia. Initial symptoms were attributed to sciatica, but further evaluation revealed a right orbital mass, gastric antral thickening, peritoneal carcinomatosis, and multiple soft tissue metastases. Biopsy of the gastric lesion confirmed high-grade, poorly differentiated adenocarcinoma, microsatellite stable, and negative for HER2 and PD-L1 expression. The patient received palliative stereotactic radiation therapy to the orbit and systemic chemotherapy with FOLFOX. Although her symptoms initially improved, she experienced multiple complications, including bacteremia, gastrointestinal bleeding, and thromboembolic events, ultimately leading to a transition to comfort-focused care. This case highlights the importance of maintaining a broad differential diagnosis in patients with persistent or atypical symptoms and underscores the need for early imaging and multidisciplinary evaluation. Recognition of rare metastatic patterns in gastric cancer is essential to avoid diagnostic delays and initiate appropriate treatment strategies that can improve quality of life, even in the setting of advanced disease.

## Introduction

Gastric adenocarcinoma remains a major contributor to cancer-related mortality worldwide, with approximately half of patients presenting with advanced disease at diagnosis [1]. Gastric adenocarcinomas are typically sporadic, with a rare genetic etiology in 5%-10%. Common risk factors include *Helicobacter pylori* infection, Epstein-Barr virus, high-salt foods, N-nitroso compounds, smoking, high consumption of smoked foods, and chronic gastroesophageal reflux. Typically, patients with gastric adenocarcinoma present initially with gastrointestinal symptoms such as indigestion, nausea, and vomiting. As the cancer progresses, symptoms include more invasive symptoms such as hematemesis, hematochezia, ascites, or palpable masses. Metastatic gastric cancer has a few well-known physical exam signs, including Virchow’s node (left supraclavicular lymphadenopathy), the Sister Mary Joseph nodule (periumbilical lymphadenopathy), and the Irish node. Paraneoplastic manifestations, including seborrheic keratosis, are occasionally observed [2]. Diagnosis is usually established by esophagogastroduodenoscopy with biopsy, along with staging CT scans. Histopathologic evaluation commonly identifies either intestinal-type or diffuse-type adenocarcinoma. In patients who are surgical candidates, perioperative chemotherapy is typically offered. In patients with advanced and non-resectable cancers, a palliative approach with radiation and chemotherapy is taken [2].

In this report, we present a case of a 48-year-old female patient with prediabetes who presented with atypical symptoms of musculoskeletal pain in the left shoulder and left lower extremity and lower back pain, along with diplopia, impaired right-eye adduction, and no gastrointestinal symptoms. In rare cases, patients may first present with signs reflecting distant metastases rather than gastrointestinal complaints. Recognizing atypical presentations is crucial to avoid diagnostic delays and initiate prompt treatment.

## Case presentation

A 48-year-old woman with a history of prediabetes presented to the clinic with a one-month history of progressively worsening pain in her lower back, left shoulder, and left lower extremity. The pain was described as deep, persistent, and occasionally radiating, interfering with her ability to sleep and perform daily activities. It was initially attributed to musculoskeletal strain or sciatica, and she was treated with non-steroidal anti-inflammatory drugs and physical therapy, but without clinical improvement. In addition, she developed new-onset diplopia during the third week of symptom progression. She denied weight loss, fever, night sweats, nausea, vomiting, early satiety, or gastrointestinal bleeding.

On physical examination, she had focal tenderness over the left paraspinal muscles and shoulder, but preserved strength and range of motion in all limbs. Neurological exam revealed impaired right-eye adduction, raising concern for a structural lesion of the extraocular muscles or cranial nerves. Given the constellation of persistent symptoms and the abnormal ocular finding, cross-sectional imaging was obtained.

**Figure 1:**
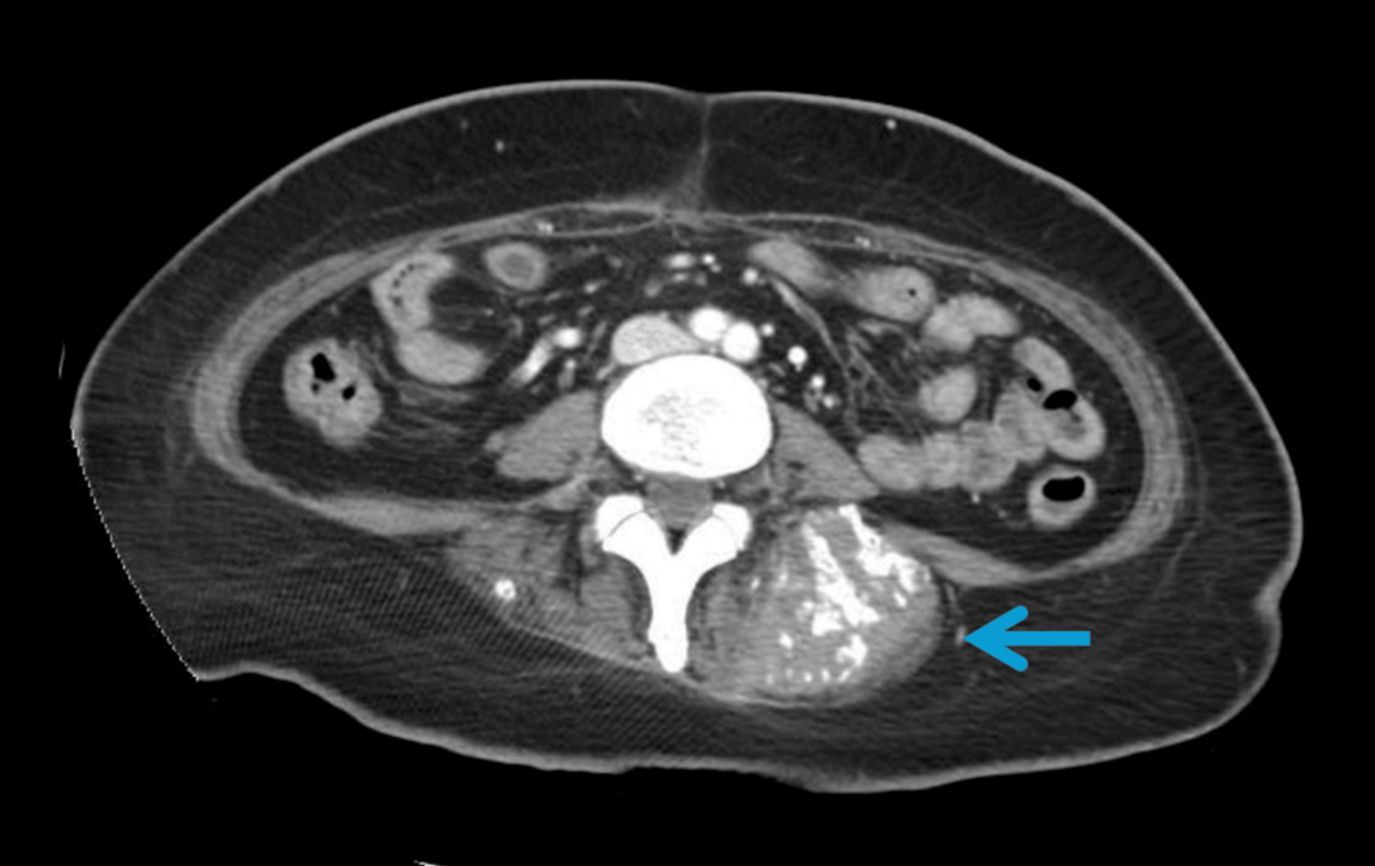
Left paraspinal musculature metastases Within the anterior intramuscular compartment of the left thigh and along the left paraspinal musculature, there are expansile, partially calcified, heterogeneous masses also compatible with intramuscular metastatic disease. Incidentally noted deep venous thrombosis of the left superficial femoral vein.

**Figure 2:**
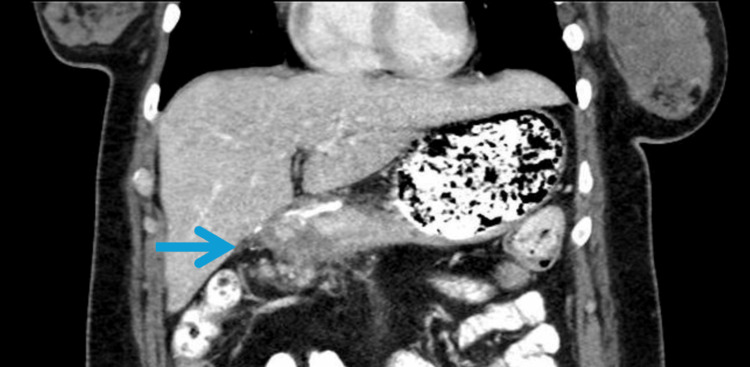
Mass-like thickening of the gastric antrum There is irregular and mass-like thickening of the gastric antrum associated with surrounding inflammatory/infiltrative changes within the mesentery. Inferiorly, there are prominent lymph nodes compatible with biopsy-proven gastric adenocarcinoma.

CT neck/chest identified fat stranding and vague hyperenhancement inferior to the left trapezius muscle, possibly representing an abscess, along with a round, mass-like lesion in the postseptal space of the right orbit. Incidental thickening of the gastric antrum prompted further abdominal imaging. CT of the abdomen and pelvis demonstrated irregular thickening of the gastric antrum with associated peritoneal implants (Figure 1), consistent with metastatic gastric cancer [2]. Multiple enhancing intramuscular lesions were also seen in the left paraspinal musculature and in the left thigh on separate imaging, concerning for metastatic disease (Figure 2) [3-5].

**Figure 3:**
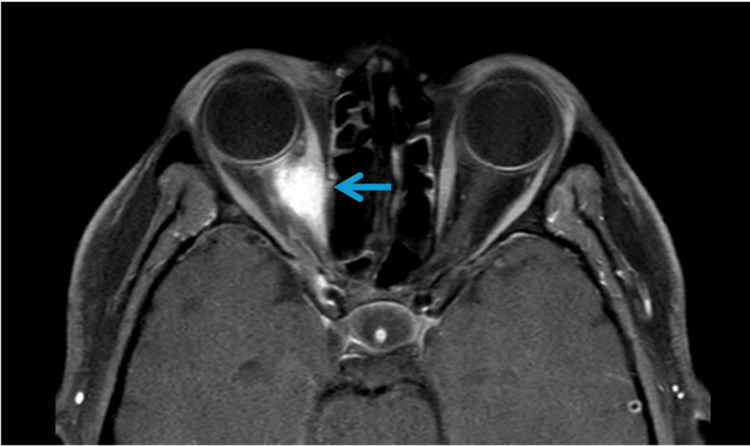
Right orbit metastases MRI orbits: within the right orbit, an enhancing mass involves the medial rectus and superior oblique and exerts mass effect on the optic nerve, resulting in deformation. Enhancement of the optic nerve suggests inflammation or infiltration.

MRI of the orbits showed an enhancing mass involving the medial rectus and superior oblique muscles with mass effect on the optic nerve (Figure 3). Orbital metastases from gastric cancer are uncommon and typically associated with aggressive disease [5,6].

Upper endoscopy revealed a friable antral mass. Biopsy confirmed high-grade, poorly differentiated adenocarcinoma, microsatellite stable, HER2-negative, and PD-L1 combined positive score < 1. Tumor markers were elevated. Next-generation sequencing did not reveal actionable mutations.

She received palliative stereotactic radiation to the orbital lesion, followed by systemic chemotherapy with FOLFOX, consistent with standard first-line cytotoxic regimens [7]. Her diplopia and pain improved briefly, but episodes of bacteremia, gastrointestinal bleeding, and thromboembolic events complicated her clinical course. Given her progressive decline, she ultimately elected for comfort-focused care.

## Discussion

Most patients with gastric adenocarcinoma present with gastrointestinal symptoms, but a subset first presents with manifestations of distant metastatic disease, which can easily lead clinicians down alternate diagnostic pathways [1,2]. This case is a clear example of how metastatic gastric cancer may initially mimic common musculoskeletal or neurologic conditions, particularly in younger or otherwise healthy individuals. The patient’s musculoskeletal pain and new-onset diplopia were initially interpreted as orthopedic and neurologic issues, which are far more common in primary care and outpatient settings. Only when symptoms persisted and evolved did imaging reveal the actual underlying cause.

Skeletal muscle metastases are uncommon, reported in less than 1% of all solid tumors, and even more rarely from gastric adenocarcinoma [3,4]. Several mechanisms have been proposed to explain this, including high tissue pressure, continuous muscle activity, fluctuating pH, and a rich microvascular network that may limit tumor implantation. The local immune microenvironment of muscle tissue may also play a protective role. Despite these factors, when muscle metastases do occur, they tend to present with non-specific symptoms such as focal pain, swelling, or limited range of motion. These features overlap significantly with benign conditions like myositis, strain, radiculopathy, or infectious myositis, contributing to delays in diagnosis.

In the limited literature available, muscle metastases from gastric cancer have been reported to involve the iliopsoas, abdominal wall, paraspinal muscles, and extremities [3,4]. Presentations have included compartment syndrome, palpable masses mistaken for abscesses, and isolated back or limb pain. The pattern seen in our patient, with involvement of both paraspinal musculature and the left thigh, is consistent with hematogenous dissemination and reflects aggressive tumor behavior.

Orbital metastasis from gastric cancer is also rare and often associated with widespread disease [5,6]. Symptoms typically include diplopia, proptosis, or orbital pain, depending on the involved structures. In our patient, involvement of the medial rectus and superior oblique muscles correlated directly with her impaired right-eye adduction. Because ocular symptoms often lead clinicians to consider neurologic or primary ophthalmologic disorders-such as cranial nerve palsy, myasthenia gravis, orbital cellulitis, or thyroid eye disease-the possibility of metastatic cancer is rarely an initial consideration.

HER2 positivity predicts response to trastuzumab, which improves survival in advanced gastric cancer [8], but as in this patient, the majority of gastric cancers are HER2-negative. The presence of both orbital and skeletal muscle metastases at diagnosis suggests a high burden of circulating tumor cells and aggressive tumor biology. These findings align with her poorly differentiated histology and lack of therapeutic biomarkers.

The prognosis of metastatic gastric cancer remains poor, particularly in patients with visceral metastases, peritoneal carcinomatosis, or high-grade tumors. Microsatellite-stable, HER2-negative, and PD-L1-negative tumors have limited responsiveness to immunotherapy and generally poorer outcomes. Cytotoxic chemotherapy with regimens such as FOLFOX or FOLFIRI remains the standard first-line approach in biomarker-negative disease [7]. While these regimens may improve symptoms and modestly extend survival, responses are often limited in duration. Focal radiation therapy can provide symptom relief, particularly for orbital or painful muscle lesions, as observed in this case. Her overall course was complicated by recurrent infections, gastrointestinal bleeding, and thromboembolic events, complications frequently encountered in advanced gastrointestinal cancers and reflective of systemic disease burden, mucosal fragility, and the prothrombotic state associated with malignancy.

## Conclusions

Clinicians should consider malignancy in patients with unexplained musculoskeletal or neurological symptoms, particularly when symptoms persist despite standard therapy. This case highlights the importance of early imaging and biopsy in atypical presentations and underscores the role of multidisciplinary care in managing advanced malignancies with uncommon metastatic patterns. Recognizing that gastric cancer can present initially with soft tissue and orbital metastases may help reduce diagnostic delays and improve the timeliness of appropriate interventions.
